# Taxonomic diversity of fungi deposited from the atmosphere

**DOI:** 10.1038/s41396-018-0160-7

**Published:** 2018-05-30

**Authors:** Cheolwoon Woo, Choa An, Siyu Xu, Seung-Muk Yi, Naomichi Yamamoto

**Affiliations:** 1 Department of Environmental Health Sciences, Graduate School of Public Health, Seoul National University, Seoul, 08826 Republic of Korea; 2 Institute of Health and Environment, Seoul National University, Seoul, 08826 Republic of Korea

**Keywords:** Biogeochemistry, Ecosystem ecology, Climate-change impacts, Microbial ecology, Molecular ecology

## Abstract

Fungi release spores into the global atmosphere. The emitted spores are deposited to the surface of the Earth by sedimentation (dry deposition) and precipitation (wet deposition), and therefore contribute to the global cycling of substances. However, knowledge is scarce regarding the diversities of fungi deposited from the atmosphere. Here, an automatic dry and wet deposition sampler and high-throughput sequencing plus quantitative PCR were used to observe taxonomic diversities and flux densities of atmospheric fungal deposition. Taxon-specific fungal deposition velocities and aerodynamic diameters (*d*_a_) were determined using a collocated cascade impactor for volumetric, particle-size-resolved air sampling. Large multicellular spore-producing dothideomycetes (*d*_a_ ≥ 10.0 μm) were predominant in dry deposition, with a mean velocity of 0.80 cm s^–1^ for all fungal taxa combined. Higher taxonomic richness was observed in fungal assemblages in wet deposition than in dry deposition, suggesting the presence of fungal taxa that are deposited only in wet form. In wet deposition, agaricomycetes, including mushroom-forming fungi, and sordariomycetes, including plant pathogenic species, were enriched, indicating that such fungal spores serve as nuclei in clouds, and/or are discharged preferentially during precipitation. Moreover, this study confirmed that fungal assemblage memberships and structures were significantly different between dry and wet deposition (*P*-test, *p* *<* 0.001). Overall, these findings suggest taxon-specific involvement of fungi in precipitation, and provide important insights into potential links between environmental changes that can disturb regional microbial communities (e.g., deforestation) and changes in precipitation patterns that might be mediated by changes in microbial communities in the atmosphere.

## Introduction

The kingdom Fungi contains several million estimated species of molds, yeasts, mushrooms, and other life forms [1, 2]. Fungi prevail in the pedosphere [3, 4], from which spores, hyphae, fragments, and other propagules are released into the global atmosphere, with an estimated annual emission of 28–50 Tg [5, 6]. The emitted fungal particles are involved in climate systems by serving as ice-forming nuclei (IN) and cloud condensation nuclei (CCN) [7–9], and/or by absorbing and reflecting solar and terrestrial radiations [10, 11]. Aerosol particles, including biological particles, are important since they provide the surface area on which ice formation and cloud condensation are catalyzed in the atmosphere. The emitted particles eventually settle on the surface of the Earth by sedimentation (dry deposition) and precipitation (wet deposition). Such aeromycological processes contribute to global biogeochemical cycles, including the nutrient and hydrological cycles [12]. Atmospheric transport is also important for microbial migration and colonization [13, 14]. Thus, fungi in the atmosphere are key components of global ecological systems.

Particle size is expected to affect flux densities of fungal deposition from the atmosphere. Aerodynamic diameter (*d*_a_) is a commonly used size measure for airborne particles. This is defined as the diameter of a sphere that has a density of 1 g cm^–3^ with the same settling velocity as the velocity of a particle of interest. Aerodynamic diameters of atmospheric fungi are taxon-dependent [15, 16], and taxon-dependent dry deposition velocities of atmospheric fungi are therefore expected, with larger velocities for large multicellular spore-producing dothideomycetes, for example. Particle size is important not only for dry deposition but also for wet deposition of biological particles. For instance, airborne particles with *d*_a_ > 2 μm serve as effective CCN that can efficiently collide and coalesce with smaller droplets, and therefore contribute to cloud precipitation [17, 18]. Indeed, literature reported that large pollen grains served as effective CCN in the atmosphere [19].

Knowledge is scarce regarding the diversities of fungi deposited from the atmosphere [20]. Petri dish trapping was traditionally used to study dry deposition. Previous studies based on analyses of settled, cultured colonies on Petri dishes containing nutrient media showed that large multicellular spore-producing dothideomycetes were abundant in dry deposition from the atmosphere in the UK [21] and Nigeria [22]. Rainwater was typically used for analysis of wet deposition [23–25]. One such study detected plant pathogenic *Fusarium* species in the atmosphere of the south-east coast of Spain using analyses of culturable fungi in rainwater samples [23]. However, these culture-based techniques are limited in their ability to detect fastidious species that are difficult to raise in culture for identification and offer limited taxonomic coverage. Previous research, for instance, reported that only 10–40% of atmospheric fungi were culturable [26], of which about 20% were sterile fungi or were *mycelia sterilia* that were not identifiable because of their slow-growing mycelia and lack of sporulation on conventional media [27]. Basidiomycetes were rarely observed in culture-based aeromycological studies [27, 28], indicating that they were not amenable to this approach. However, Hassett et al. [7] showed that basidiospores formed effective cloud nuclei, highlighting the need for accurate atmospheric detection methods that encompass non-culturable species.

The goal of this study was to examine taxonomic diversity of fungi deposited from the atmosphere. Specifically, we analyzed differences in fungal assemblages between dry and wet deposition. Here, we used DNA-based methods to analyze atmospheric fungal species regardless of cultivability. These culture-independent methods can thus reveal accurate pictures of the atmospheric fungal assemblages, including basidiomycetes [15, 29], which are thought to play significant roles in global ecological systems.

## Materials and methods

### Dry and wet deposition sampling

Dry and wet deposition samples were collected using a custom-made automatic dry and wet deposition sampler [30] (Supplementary Fig. S1) on the roof (~20 m above ground) of a building in Seoul in South Korea (37°27′55.0″N; 126°57′17.7″E), which is situated in the humid continental climate zone, from May to November 2015. The sampling point is surrounded by the hilly, forested area of the southern outskirts of the Seoul Special Metropolitan City. Ambient temperature, relative humidity, wind velocity, and precipitation during the sampling periods are presented in Supplementary Table S1. Each of the dry and wet deposition samplings was done in duplicate and continued for 1 month. The dry deposition samples were collected on 47 mm diameter quartz fiber substrates (QR-100; Advantec Tokyo Kaisha, Ltd, Tokyo, Japan) with its surface coated with 1 mL mineral oil (CAS no. 8042-47-5; Daejung Chemicals & Metals Co., Ltd, Siheung, South Korea) to minimize particle re-entrainment. Each of the wet deposition samples was collected in a polypropylene funnel (with its effective collection area of 167 cm^2^) connected to a 1 L polypropylene bottle by a Teflon adaptor, recovered immediately after each precipitation that typically continued for one to several days, and stored in 250 mL polypropylene bottle(s). In total, 14 dry and 14 wet deposition samples were collected (Supplementary Table S2). The samples were kept at −20 °C until DNA extraction.

### Air sampling

Each of the air samples was collected for a duration of 1 month and co-located on the same roof for deposition samplings. The sampling in August failed due to an intense rainfall event. An eight-stage non-viable Andersen sampler (AN-200; Sibata Scientific Technology Ltd, Tokyo, Japan) was used to collect airborne particles on 80 mm diameter glass fiber substrates (QR-100; Advantec Tokyo Kaisha, Ltd, Tokyo, Japan) with an air flow rate at 28.3 L min^−1^. No mineral oil was applied onto collection substrates for air sampling. The cutoff aerodynamic diameters at 50% collection efficiency were 0.43–0.65, 0.65–1.1, 1.1–2.1, 2.1–3.3, 3.3–4.7, 4.7–7.0, 7.0–11, and >11 μm. Substrates loaded to the stages of *d*_a_ = 0.43–0.65, 0.65–1.1, and 1.1–2.1 μm were not analyzed due to inadequate biomass [15]. A total of 30 air samples were collected (Supplementary Table S2). The samples were kept at −20 °C until DNA extraction.

### DNA extraction

DNA was extracted using a protocol reported elsewhere [31]. For dry deposition samples, one quarter fraction of each sampled substrate was used for DNA extraction. Each wet deposition sample was filtered to recover particulate matters, including fungal spores, onto a 47 mm diameter filter using a sterile MiroFunnelTM Filter Funnel (0.45 µm pore size, GN-6 Mericel^®^ white gridded membrane; Pall Corporation, NY, USA) of which one quarter area fraction was used for DNA extraction. One-eighth area fraction of each air sample substrate was used for DNA extraction. A PowerMax^®^ Soil DNA Isolation Kit (Mobio Laboratory, CA, USA) was used with additional 0.1 mm diameter glass beads (300 mg) and 0.5 mm diameter glass beads (100 mg) for 3 min sample homogenization by a bead beater (BioSpec Products, OK, USA). After homogenization, DNA was extracted, purified, and eluted into 30 μL TE (10 mM Tris–HCl, 1 mM EDTA, pH = 8.0).

### DNA sequencing

DNA libraries were constructed according to a protocol reported elsewhere [32]. Briefly, the fungal internal transcribed spacer 1 (ITS1) region was amplified with universal fungal primers ITS1F and ITS2 [33, 34] along with adapter sequences for Illumina MiSeq. One of the duplicates was not PCR-amplifiable in three of seven dry deposition samples, resulting in four of seven pairs of dry and wet deposition sample duplicates available for subsequent analyses. The purified amplicons were indexed using the Nextera XT Index kit (Illumina, Inc., CA, USA). Each of the purified indexed amplicons was normalized to 4 nM in 10 mM Tris–HCl (pH = 8.5) and pooled with an internal control PhiX (30%). The pooled, heat-denatured amplicons were loaded to a v3 600 cycle-kit reagent cartridge (Illumina), and 2 × 300 bp paired-end sequencing was performed by Illumina MiSeq. Raw sequence data were submitted to the NCBI Sequence Read Archive under project number SRP119277.

### DNA sequence processing and analyses

The full sequence processing protocol is available elsewhere [32]. Briefly, the paired-end reads were joined with a minimum allowed overlap of 10 bp in QIIME version 1.8.0 [35]. Chimeric sequences were excluded using the chimera.vsearch command [36] in mothur v.1.39.5 [37] against the uchime_reference_dataset_ITS1_28.06.2017.fasta database [38]. A total of 960,088 high-quality sequences were obtained from 55 libraries, with mean lengths ranging from 267 to 318 bp (Supplementary Table S2). Each of the resultant sequences were taxonomically assigned using BLASTN2.2.28+ against the UNITEdatabaseinFHiTINGSformat20-11-2016release.fasta database and classified using FHiTHINGS version 1.4 [39]. In addition, each sequence was BLASTN-searched against the latest version of the fasta file containing sequences of unidentified species hypotheses, i.e., top50_release_01.12.2017.fasta [40], for the possible presence of such rare species in the atmosphere. For taxonomic assignment, the sequences were not clustered into operational taxonomic units (OTUs), since no consensus threshold was available for fungal ITS [41]. For diversity analyses, the sequences were clustered into OTUs at 97% sequence similarity [4, 15]. For the α diversity analysis, the numbers of observed OTUs were determined based on 4000 sequences subsampled from each library. The β diversity analyses were conducted based on Jaccard indices and Yue and Clayton theta similarity coefficients as measures of fungal assemblage memberships and structures, respectively. *P*-test analysis showed that the variability in characterizing the fungal assemblage structures within a sample duplicate was significantly smaller than the differences across the samples (ParsScore = 13, *p* *=* 0.0421) (Supplementary Fig. S2a).

### Quantitative PCR

Quantitative PCR (qPCR) was performed to quantify gene copy numbers (GCN) of fungal ITS1 using a method reported elsewhere [32]. The same primers were used for sequencing (ITS1F and ITS2). Briefly, each of the 20 μL reaction mixtures included 1× Fast SYBR Green Master mix reagent (Clontech Laboratories, Inc., CA, USA), 10 μM forward and reverse primers, and 1 μL template DNA. Quantitative PCR was performed using a 7300 Real-Time PCR System (Applied Biosystems, Inc., CA, USA). Standard curves were generated against known concentrations of ITS1 amplicons by conventional PCR with the primers ITS1F/ITS2 and a template DNA extracted from *Aspergillus fumigatus* ATCC MYA4609. Each assay was in triplicate. No inhibition was found, in line with the previously reported method [31]. Briefly, no inhibition was confirmed in that we observed no significant difference in quantitated results of 10^6^ GCN *A. fumigatus* DNA standard with and without spiking 1 μL DNA extract from each of air or deposition samples in question. Extraction efficiency of DNA was assumed to be 10% [31]. The reproducibility of measurements of fungal concentrations in biologically duplicated dry and wet deposition samples is shown in Supplementary Fig. S2b. Reproducibility was assessed in terms of cumulative coefficient of variation [42].

### Data processing and statistical analyses

Taxon-specific fungal concentration was determined by multiplying the sequencing-derived relative abundance of a taxon by the universal fungal qPCR-derived total fungal concentration [16, 32, 43]. Geometric mean of aerodynamic diameter (*d*_g_) of a given particle size distribution was computed by using the GM calculator version 1.0 [16]. Arithmetic mean of *d*_g_ of all sampled months was used as a representative *d*_g_ for each taxon. The dry deposition velocity (*V*_d_) was calculated by the following equation:

1 $$V_{\mathrm{d}}\,=\,\mathop {\sum}\limits_{j = 1}^6 {\mathop {\sum}\limits_{i = 1}^5 {N_{j,i}} } {{\Bigg / }}\mathop {\sum}\limits_{j = 1}^6 {F_j}$$

where *N*_*j,i*_ is the airborne fungal concentration (GCN m^–3^) in the *i*th particle size interval measured for the *j*th month by the Andersen sampler, and *F*_*j*_ is the dry deposition flux density (GCN cm^–2^ month^–1^) measured for the *j*th month by the dry deposition sampler. August 2015 data were excluded as air sampling failed. The calculated dry deposition velocities were compared with the Stokes terminal gravitational settling velocities (*V*_Stk_) given by the following equation:

2 $$V_{{\mathrm{Stk}}}{\mathrm{ = }}\frac{{\rho _0d_{\mathrm{a}}^2g}}{{{\mathrm{18}}\eta }}$$

where *ρ*_0_ is the standard particle density (=1.0 g cm^−3^), *g* is the acceleration of gravity (=980 cm s^−2^), and *η* is the viscosity of air (=1.8 × 10^−5^ Pa s). The comparison of the calculated dry deposition velocity with the Stokes velocity gives insights into behaviors and fates (e.g., removal by wet deposition) of fungal particles in the atmosphere.

Mothur v.1.39.5 was used to perform *P*-tests to compare fungal assemblage structures and memberships. SAS version 9.4 (SAS Institute Inc., NC, USA) was used for a paired *t*-test to compare the taxonomic richness between dry and wet deposition.

## Results

### Air

**Fig. 1:**
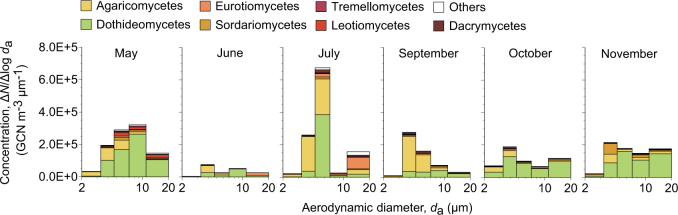
Particle size distributions of atmospheric fungal concentrations, in terms of gene copy number (GCN) of fungal ITS1, measured by the Andersen sampler in Seoul in South Korea from May to November 2015. Monthly results are shown, except for August when sampling failed

More than 99% of the sequences from air samples were found to represent the Ascomycota and the Basidiomycota, with the relative abundance of Ascomycota increasing with increasing aerodynamic diameter, i.e., 48, 47, 67, 87, and 89% for *d*_a_ = 2.1–3.3, 3.3–4.7, 4.7–7.0, 7.0–11, and >11 μm, respectively. The two most abundant classes were Dothideomycetes and Agaricomycetes (Fig. 1), with mean annual concentrations of 71,000 and 35,000 GCN m^–3^, respectively. Dothideomycete particles were larger than Agaricomycete particles, with geometric means of aerodynamic diameters (*d*_g_) of 7.32 and 4.34 μm, respectively.

**Fig. 2:**
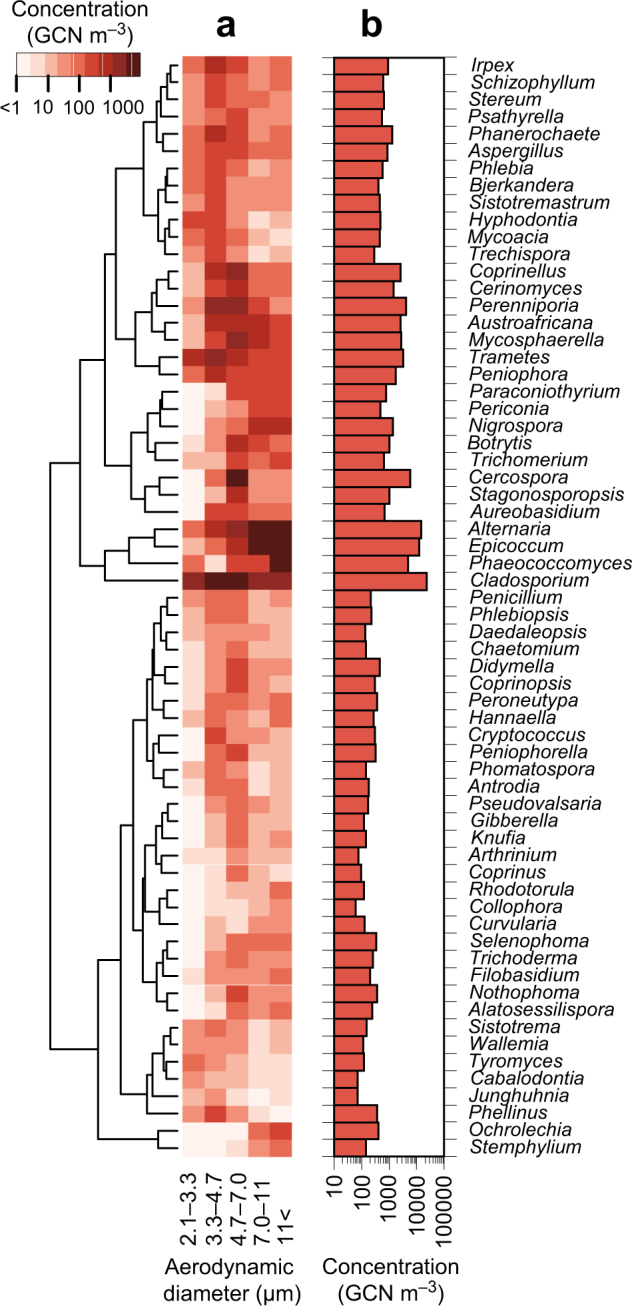
Annual mean concentrations of selected fungal genera. Genera listed in Supplementary Table S3 are shown. Taxonomic information of the genera shown is available in Supplementary Table S4. **a** Particle size-resolved concentrations. The tree represents between-genus similarity of the log-transformed concentrations based on Euclidean distance. **b** Particle size-integrated concentrations

The *d*_g_ values of selected genera are listed in Supplementary Table S3, and their taxonomic information is available in Supplementary Table S4. Genera were shown if they were detected in all sampled months and each month had more than four sequence reads from the samples of all particle size intervals combined. *Rhodotorula* was not detected in air samples collected in June, but was included due to its importance in dry and wet deposition (see the next section). The concentrations of selected genera are shown in Fig. 2.

A total of 131 sequences from the air samples were matched with the sequences of four unidentified species hypotheses in the list of the top 50 most wanted fungi with the alignment length longer than 250 bp and 100% identity (Supplementary Table S5). These species hypotheses were SH027064.07FU, SH468151.07FU, SH455726.07FU, and SH459716.07FU.

### Dry and wet deposition

**Fig. 3:**
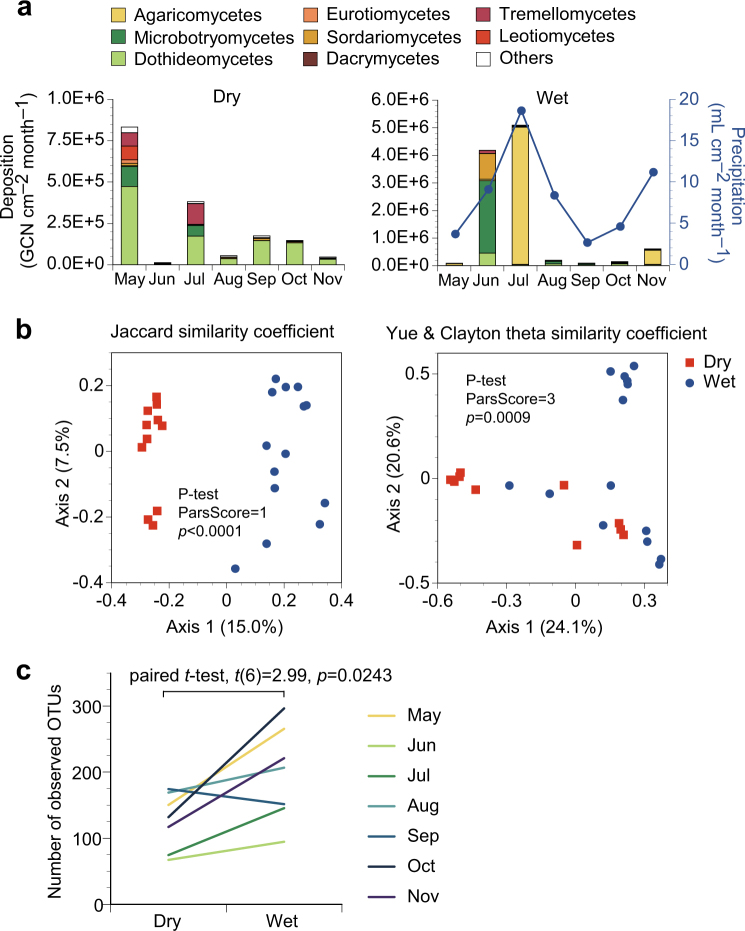
Dry and wet deposition of atmospheric fungi. **a** Flux densities of monthly dry and wet fungal deposition and precipitation are shown. Data are averaged from duplicate measurements. **b** Principal coordinate analysis plots for fungal assemblage memberships (Jaccard similarity coefficients) and structures (Yue and Clayton theta similarity coefficients) based on ITS1 OTUs at 97% sequence similarity. Each data point represents an individual sample duplicate collected for each month. **c** Taxonomic richness in terms of the numbers of observed OTUs from 4000 sequences subsampled from each library. Data from the same month are connected by a line. The mean value is shown for each of the duplicate measurements

More than 99% of the sequences from deposition samples were found to represent the Ascomycota and the Basidiomycota, with a larger relative abundance of Ascomycota in dry deposition (84%) than in wet deposition (35%). The annual mean flux densities of dry and wet deposition were 240,000 and 1,490,000 GCN cm^–2^ month^–1^, respectively, for all fungal taxa combined. Dothideomycetes, Tremellomycetes, and Microbotryomycetes were the three most abundant classes in dry deposition (Fig. 3a), with mean flux densities of 142,000, 30,000, and 27,000 GCN cm^–2^ month^–1^, respectively. Agaricomycetes, Microbotryomycetes, and Sordariomycetes were the three predominant classes in wet deposition (Fig. 3a), with mean flux densities of 804,000, 401,000, and 144,000 GCN cm^–2^ month^–1^, respectively. Figure 3b shows principal coordinate analysis plots of fungal assemblage memberships and structures based on OTUs of fungal ITS1 at 97% sequence similarity, demonstrating that fungal assemblage memberships (*P*-test, ParsScore = 1, *p* *<* 0.0001) and structures (*P*-test, ParsScore = 3, *p* *=* 0.0009) were significantly different between dry and wet deposition. Taxonomic richness in terms of the numbers of observed OTUs was higher for wet deposition (paired *t*-test, *t*(6) = 2.99, *p* = 0.0243) (Fig. 3c).

**Fig. 4:**
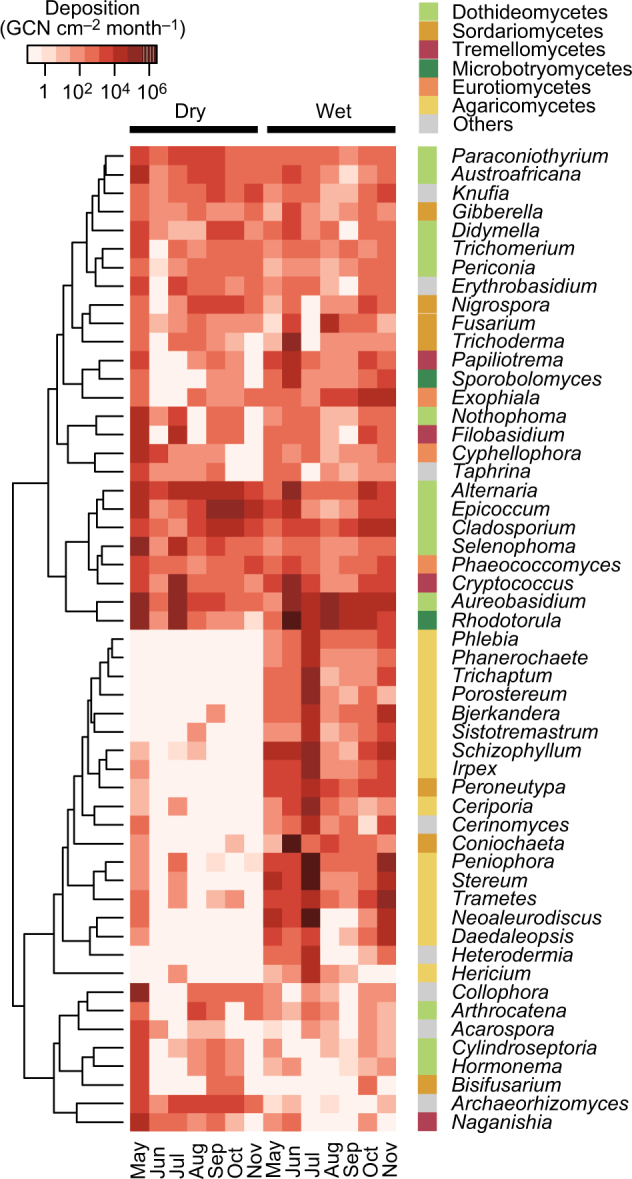
Deposition flux densities of the 30 most abundant fungal genera detected from dry or wet deposition samples. The tree represents between-genus similarity of the log-transformed deposition flux densities based on Euclidean distance. Taxonomic information of the genera shown is available in Supplementary Table S4

Figure 4 shows the flux densities of the 30 most abundant genera in dry or wet deposition samples. Dothideomycetes including *Alternaria*, *Aureobasidium*, and *Epicoccum* were abundant in both dry and wet deposition, with similar tendencies observed for *Cryptococcus* and *Rhodotorula* in the Microbotryomycete and Tremellomycete classes, respectively. Agaricomycetes such as *Peniophora*, *Stereum*, and *Schizophyllum* were enriched only in wet deposition, with a similar tendency observed for *Peroneutypa* and *Coniochaeta* sordariomycetes.

A total of 1167 sequences from the dry and wet deposition samples were matched with the sequences of three unidentified species hypotheses in the list of the top 50 most wanted fungi with the alignment length longer than 250 bp and 100% identity (Supplementary Table S5). These species hypotheses were SH493298.07FU, SH468151.07FU, and SH455726.07FU.

### Dry deposition velocities

**Fig. 5:**
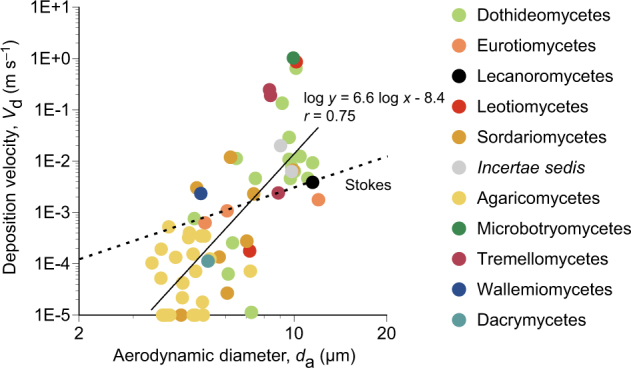
Dry deposition velocities of selected fungal genera as a function of aerodynamic diameters. Each point represents a genus. Genera listed in Supplementary Table S3 are displayed

Figure 5 shows dry deposition velocities, calculated according to Eq. 1 in the Method section above, as a function of aerodynamic diameters for the selected fungal genera (Supplementary Table S3). Larger deposition velocities were associated with larger aerodynamic diameters, with a Pearson’s correlation coefficient of 0.75. The genera with larger aerodynamic diameters tended to settle faster than the Stokes velocities, while the genera with smaller aerodynamic diameters tended to settle slower than the Stokes velocities. The mean settling velocity was 0.80 cm s^–1^ for all fungal taxa combined.

## Discussion

Two classes of fungi were predominant in air: Dothideomycetes and Agaricomycetes (Fig. 1). *Perenniporia* and *Trametes* were the two most abundant agaricomycetes (Fig. 2). Owing to identification difficulty, these mushroom-forming basidiomycetes were reported rarely in previous culture-based aeromycological studies [27, 28]. From the air and deposition samples, we also detected five unidentified species hypotheses in the list of the top 50 most wanted fungi [40] (Supplementary Table S5), suggesting the atmosphere as an ideal melting pot, due to its large entropy of mixing, where such rare species can be studied in conjunction with sensitive high-throughput sequencing techniques. *Cladosporium*, *Alternaria*, and *Epicoccum* were the three most abundant dothideomycetes, with 23,000, 15,000, and 13,000 GCN m^–3^, respectively, with higher concentrations than other common airborne genera (e.g., 870 GCN m^–3^ for *Aspergillus* and 220 GCN m^–3^ for *Penicillium*) (Fig. 2). *Alternaria* and *Epicoccum* form multicellular spores, while *Aspergillus* and *Penicillium* form unicellular spores. The high dothideomycete concentrations, in terms of ITS1 GCN, might be partly due to the multicellularity of their spores, which yield large numbers of per-spore ITS copies.

Large spore-producing dothideomycetes were predominant in dry deposition (Fig. 3a), with examples including *Alternaria*, *Aureobasidium*, and *Epicoccum* (Fig. 4). *Alternaria* and *Epicoccum* form spores with reported microscopy-based sizes of 18–83 × 7–18 and 15–25 μm, respectively [44]. *Aureobasidium* produces chlamydospores with a reported length of 17 μm [45]. Dry deposition velocities and aerodynamic diameters were 0.63 cm s^–1^ for *Alternaria* (*d*_g_ = 10.0 μm), 13 cm s^–1^ for *Aureobasidium* (*d*_g_ = 9.14 μm), and 0.93 cm s^–1^ for *Epicoccum* (*d*_g_ = 11.5 μm) (Supplementary Table S3). Smaller velocities were observed for other common airborne genera, with examples of 0.063 cm s^–1^ for *Aspergillus* (*d*_g_ = 5.13 μm) and 0.11 cm s^–1^ for *Penicillium* (*d*_g_ = 6.05 μm), suggesting that larger dothideomycete spores settled faster than smaller eurotiomycete spores due to a larger gravitational influence. Moreover, dothideomycetes tended to settle faster than the Stokes velocities (Fig. 5), indicating that their motions were influenced not only by gravity but by additional forces, such as inertia due to atmospheric turbulence [46].

Small spore sizes are reported for many agaricomycetes, with examples of 6.5–8 × 3–3.5 μm for *Peniophora* [47], 6–7 × 2.5 μm for *Stereum* [48], and 6.6–9.2 × 2.4–3 μm for *Trametes* [49]. Their aerodynamic diameters were similarly small and were associated with small settling velocities (Fig. 5), for example, 0.034 cm s^–1^ for *Peniophora* (*d*_g_ = 5.12 μm), 0.032 cm s^–1^ for *Stereum* (*d*_g_ = 4.54 μm), and 0.013 cm s^–1^ for *Trametes* (*d*_g_ = 4.12 μm) (Supplementary Table S3). The measured velocities were smaller than the Stokes velocities (Fig. 5), indicating that particles dissipated from the atmosphere before they settled in the sampler. This is expected due to their long residence times in the atmosphere because of the small settling velocities. One possibility is that the spores were removed by attachment onto surfaces of the surrounding objects, such as trees or buildings [50]. Another possibility is that spores were removed by wet deposition, such as washout (below-cloud scavenging) and rainout (in-cloud scavenging) [51].

Mushroom-forming agaricomycetes were enriched in wet deposition but not in dry deposition, with a similar tendency observed for the sordariomycete genera *Peroneutypa* and *Coniochaeta* (Fig. 4), suggesting that these were scavenged into raindrops by washout and/or rainout mechanisms due to, e.g., IN and/or CCN activities [7, 9, 52, 53]. Another possibility is that such fungal spores were discharged preferentially during precipitation. Indeed, agaricomycetes comprise a group of fungi that actively discharge spores primarily under humid conditions [5], including *Stereum*, *Trametes*, and *Schizophyllum* [54–56]. *Peroneutypa* has a closely related sordariomycete genus *Eutypella* [57] whose ascospores are discharged only when rainfall occurs [58]. Our observation suggests two non-mutually exclusive hypotheses: (1) spores serve as nuclei in clouds, and/or (2) spores are released selectively during precipitation. The causality is unknown, however. More research is thus needed to dissect the causal relationships and potential feedback mechanisms.

Large multicellular spore-producing dothideomycetes, such as *Alternaria* and *Epicoccum* were enriched in both dry and wet deposition, with a similar tendency observed for the yeast or yeast-like organisms of *Rhodotorula* and *Cryptococcus* (Fig. 4). Reported microscopy-based cell sizes for these yeast or yeast-like organisms are 3–5 µm [59] and 5–10 μm [60], respectively. Smaller size was reported for spores of *Cryptococcus neoformans* (1–2 μm) [60]. Measured aerodynamic diameters were larger, i.e., 9.94 µm for *Rhodotorula*, and 8.32 μm for *Cryptococcus* (Supplementary Table S3), indicating that these yeast or yeast-like organisms formed aggregates and/or attached to abiotic particles in the atmosphere. Large biological particles such as pollen grains are known to serve as giant CCN (GCCN) that can efficiently coalesce with smaller droplets and thus contribute to cloud precipitation [17, 18]. Our results indicate that large-sized fungal spores and yeast aggregates may similarly serve as GCCN in the atmosphere.

Aerodynamic diameter was found to strongly influence fungal deposition from the atmosphere (e.g., Fig. 5). Additional factors may include IN activities associated with proteins produced, for instance, by the plant pathogenic bacterium *Pseudomonas syringae* [61]. *P. syringae* serves as IN in the atmosphere and plays a significant role in the Earth’s hydrological cycle [62]. IN activities were previously reported for the fungal genus *Fusarium* [52, 63–66], which includes plant pathogenic species [67]. *Fusarium* was detected from wet deposition, along with the other sordariomycete genera *Coniochaeta* and *Peroneutypa* (Fig. 4). *Coniochaeta* comprises of pathogenic species of trees [68], whereas *Peroneutypa* is closely related to the genus *Eutypella* [57] that includes plant pathogenic species [69]. The enrichment of plant pathogenic fungi in wet deposition may reflect evolutionary parallelisms between bacteria and fungi in their strategies of dispersal and maintenance in the atmosphere to survive in the environment as plant pathogenic microorganisms.

Fungal assemblage memberships and structures differed significantly between dry and wet deposition (*P*-test, *p* *<* 0.001) (Fig. 3b), indicating taxon-specific involvement of fungi in the Earth’s precipitation. The finding provides important insights into relationships between environmental changes and changes in precipitation patterns that are potentially mediated by microbial communities in the atmosphere. Atmospheric fungi originate from the pedosphere. This indicates that changes in terrestrial fungal communities, for example, by human-induced land-use changes (e.g., deforestation), can cause changes in the atmospheric fungal assemblages [70]. A hypothesis can be made that deforestation reduces the population of forest-obligate basidiomycetes (e.g., mushrooms) that discharge nucleating active basidiospores responsible for cloud formation and precipitation, resulting in a negative feedback loop of drought and deforestation [7]. At present, little is known about how such environmental changes disturb Earth’s biogeochemical cycles by changing the atmospheric fungal communities. More research, with additional sampling sites to test geographical reproducibility, is thus needed to elucidate such feedback mechanisms potentially mediated by microbial communities in the atmosphere.

## Electronic supplementary material

Supplementary Information

## References

[1] Hawksworth DL, Lücking R, Heitman J, Howlett BJ, Crous PW, Stukenbrock EH, James TY, Gow NAR (2017). Fungal diversity revisited: 2.2 to 3.8 million species. The fungal kingdom.

[2] Taylor DL, Hollingsworth TN, McFarland JW, Lennon NJ, Nusbaum C, Ruess RW (2014). A first comprehensive census of fungi in soil reveals both hyperdiversity and fine‐scale niche partitioning. Ecol Monogr.

[3] Tedersoo L, Bahram M, Põlme S, Kõljalg U, Yorou NS, Wijesundera R (2014). Global diversity and geography of soil fungi. Science.

[4] Buée M, Reich M, Murat C, Morin E, Nilsson RH, Uroz S (2009). 454 pyrosequencing analyses of forest soils reveal an unexpectedly high fungal diversity. New Phytol.

[5] Elbert W, Taylor PE, Andreae MO, Pöschl U (2007). Contribution of fungi to primary biogenic aerosols in the atmosphere: wet and dry discharged spores, carbohydrates, and inorganic ions. Atmos Chem Phys.

[6] Heald CL, Spracklen DV (2009). Atmospheric budget of primary biological aerosol particles from fungal spores. Geophys Res Lett.

[7] Hassett MO, Fischer MWF, Money NP (2015). Mushrooms as rainmakers: how spores act as nuclei for raindrops. PLoS One.

[8] Pöschl U, Martin ST, Sinha B, Chen Q, Gunthe SS, Huffman JA (2010). Rainforest aerosols as biogenic nuclei of clouds and precipitation in the Amazon. Science.

[9] Haga DI, Burrows SM, Iannone R, Wheeler MJ, Mason RH, Chen J (2014). Ice nucleation by fungal spores from the classes *Agaricomycetes*, *Ustilaginomycetes*, and *Eurotiomycetes*, and the effect on the atmospheric transport of these spores. Atmos Chem Phys.

[10] Spänkuch D, Döhler W, Güldner J (2000). Effect of coarse biogenic aerosol on downwelling infrared flux at the surface. J Geophys Res-Atmos.

[11] Guyon P, Graham B, Roberts GC, Mayol-Bracero OL, Maenhaut W, Artaxo P (2004). Sources of optically active aerosol particles over the Amazon forest. Atmos Environ.

[12] Després VR, Huffman JA, Burrows SM, Hoose C, Safatov AS, Buryak G (2012). Primary biological aerosol particles in the atmosphere: a review. Tellus Ser B-Chem Phys Meteorol.

[13] Itani GN, Smith CA (2016). Dust rains deliver diverse assemblages of microorganisms to the Eastern Mediterranean. Sci Rep.

[14] Peter H, Hörtnagl P, Reche I, Sommaruga R (2014). Bacterial diversity and composition during rain events with and without Saharan dust influence reaching a high mountain lake in the Alps. Environ Microbiol Rep.

[15] Yamamoto N, Bibby K, Qian J, Hospodsky D, Rismani-Yazdi H, Nazaroff WW (2012). Particle-size distributions and seasonal diversity of allergenic and pathogenic fungi in outdoor air. ISME J.

[16] Yamamoto N, Nazaroff WW, Peccia J (2014). Assessing the aerodynamic diameters of taxon-specific fungal bioaerosols by quantitative PCR and next-generation DNA sequencing. J Aerosol Sci.

[17] Johnson DB (1982). The role of giant and ultragiant aerosol particles in warm rain initiation. J Atmos Sci.

[18] Möhler O, DeMott PJ, Vali G, Levin Z (2007). Microbiology and atmospheric processes: the role of biological particles in cloud physics. Biogeosciences.

[19] Pope FD (2010). Pollen grains are efficient cloud condensation nuclei. Environ Res Lett.

[20] Chen W, Hambleton S, Seifert KA, Cariose O, Diarra MS, Peters RD et al. Assessing performance of spore samplers in monitoring aeromycobiota and fungal plant pathogen diversity in Canada. Appl Environ Microbiol. 2018. 10.1128/aem.02601-02617. 10.1128/AEM.02601-17 PMC5930333 29475862

[21] Pawsey RG, Heath LAF (1964). An investigation of the spore population of the air at Nottingham. Trans Br Mycol Soc.

[22] Dransfield M (1966). The fungal air-spora at Samaru, Northern Nigeria. Trans Br Mycol Soc.

[23] Palmero D, Rodríguez JM, de Cara M, Camacho F, Iglesias C, Tello JC (2011). Fungal microbiota from rain water and pathogenicity of *Fusarium* species isolated from atmospheric dust and rainfall dust. J Ind Microbiol Biotechnol.

[24] Bauer H, Kasper-Giebl A, Löflund M, Giebl H, Hitzenberger R, Zibuschka F (2002). The contribution of bacteria and fungal spores to the organic carbon content of cloud water, precipitation and aerosols. Atmos Res.

[25] Kolby JE, Ramirez SD, Berger L, Griffin DW, Jocque M, Skerratt LF (2015). Presence of amphibian chytrid fungus (*Batrachochytrium dendrobatidis*) in rainwater suggests aerial dispersal is possible. Aerobiologia.

[26] Palmgren U, Ström G, Blomquist G, Malmberg P (1986). Collection of airborne micro-organisms on Nuclepore filters, estimation and analysis—CAMNEA method. J Appl Bacteriol.

[27] Takahashi T (1997). Airborne fungal colony-forming units in outdoor and indoor environments in Yokohama, Japan. Mycopathologia.

[28] Shelton BG, Kirkland KH, Flanders WD, Morris GK (2002). Profiles of airborne fungi in buildings and outdoor environments in the United States. Appl Environ Microbiol.

[29] Adams RI, Miletto M, Taylor JW, Bruns TD (2013). Dispersal in microbes: fungi in indoor air are dominated by outdoor air and show dispersal limitation at short distances. ISME J.

[30] Han JS, Seo YS, Kim MK, Holsen TM, Yi SM (2016). Total atmospheric mercury deposition in forested areas in South Korea. Atmos Chem Phys.

[31] Hospodsky D, Yamamoto N, Peccia J (2010). Accuracy, precision, and method detection limits of quantitative PCR for airborne bacteria and fungi. Appl Environ Microbiol.

[32] An C, Woo C, Yamamoto N (2018). Introducing DNA-based methods to compare fungal microbiota and concentrations in indoor, outdoor, and personal air. Aerobiologia.

[33] Gardes M, Bruns TD (1993). ITS primers with enhanced specificity for Basidiomycetes: application to the identification of mycorrhizae and rusts. Mol Ecol.

[34] White TJ, Bruns T, Lee S, Taylor J, Innis MA, Gelfand DH, Sninsky JJ, White TJ (1990). Amplification and direct sequencing of fungal ribosomal RNA genes for phylogenetics. PCR protocols a guide to methods and applications..

[35] Caporaso J, Kuczynski J, Stombaugh J, Bittinger K, Bushman F, Costello E (2010). QIIME allows analysis of high-throughput community sequencing data. Nat Methods.

[36] Rognes T, Flouri T, Nichols B, Quince C, Mahé F (2016). VSEARCH: a versatile open source tool for metagenomics. PeerJ.

[37] Schloss PD, Westcott SL, Ryabin T, Hall JR, Hartmann M, Hollister EB (2009). Introducing mothur: open-source, platform-independent, community-supported software for describing and comparing microbial communities. Appl Environ Microbiol.

[38] Nilsson RH, Tedersoo L, Ryberg M, Kristiansson E, Hartmann M, Unterseher M (2015). A comprehensive, automatically updated fungal ITS sequence dataset for reference-based chimera control in environmental sequencing efforts. Microbes Environ.

[39] Dannemiller K, Reeves D, Bibby K, Yamamoto N, Peccia J (2014). Fungal high-throughput taxonomic identification tool for use with next-generation sequencing (FHiTINGS). J Basic Microbiol.

[40] Nilsson RH, Wurzbacher C, Bahram M, R. M. Coimbra V, Larsson E, Tedersoo L (2016). Top 50 most wanted fungi. MycoKeys.

[41] Yamamoto N, Bibby K (2014). Clustering of fungal community internal transcribed spacer sequence data obscures taxonomic diversity. Environ Microbiol.

[42] Hyslop NP, White WH (2009). Estimating precision using duplicate measurements. J Air Waste Manag Assoc.

[43] Dannemiller K, Lang-Yona N, Yamamoto N, Rudich Y, Peccia J (2014). Combining real-time PCR and next-generation DNA sequencing to provide quantitative comparisons of fungal aerosol populations. Atmos Environ.

[44] Cole GT, Samson RA, Al-Doory Y, Domson JF (1984). The conidia. Mould allergy.

[45] Wachowska U, Głowacka K, Mikołajczyk W, Kucharska K (2016). Biofilm of *Aureobasidium pullulans* var. *pullulans* on winter wheat kernels and its effect on other microorganisms. Microbiology.

[46] Kim E, Kalman D, Larson T (2000). Dry deposition of large, airborne particles onto a surrogate surface. Atmos Environ.

[47] Whelden RM (1936). A comparative study of basidia and cystidia in *Peniophora livida*. Am J Bot.

[48] Burt EA (1920). The Thelephoraceae of North America. XII. Ster Ann Mo Bot Gard.

[49] Li HJ, Cui BK (2010). A new *Trametes* species from Southwest China. Mycotaxon.

[50] Nowak DJ, Hirabayashi S, Bodine A, Hoehn R (2013). Modeled PM_2.5_ removal by trees in ten U.S. cities and associated health effects. Environ Pollut.

[51] Kajino M, Aikawa M (2015). A model validation study of the washout/rainout contribution of sulfate and nitrate in wet deposition compared with precipitation chemistry data in Japan. Atmos Environ.

[52] Pouleur S, Richard C, Martin JG, Antoun H (1992). Ice nucleation activity in *Fusarium acuminatum* and *Fusarium avenaceum*. Appl Environ Microbiol.

[53] Fröhlich-Nowoisky J, Burrows SM, Xie Z, Engling G, Solomon PA, Fraser MP (2012). Biogeography in the air: fungal diversity over land and oceans. Biogeosciences.

[54] Fischer MWF, Stolze-Rybczynski JL, Cui Y, Money NP (2010). How far and how fast can mushroom spores fly? Physical limits on ballistospore size and discharge distance in the Basidiomycota. Fungal Biol.

[55] Zoberi MH (1964). Effect of temperature and humidity on ballistospore discharge. Trans Br Mycol Soc.

[56] Dye MH (1974). Basidiocarp development and spore release by *Stereum purpureum* in the field. New Zeal J Agr Res.

[57] Mayorquin JS, Wang DH, Twizeyimana M, Eskalen A (2016). Identification, distribution, and pathogenicity of Diatrypaceae and Botryosphaeriaceae associated with citrus branch canker in the Southern California desert. Plant Dis.

[58] Lachance D (1971). Discharge and germination of *Eutypella parasitica* ascospores. Can J Bot.

[59] Hernández-Almanza A, Cesar Montanez J, Aguilar-González MA, Martínez-Ávila C, Rodríguez-Herrera R, Aguilar CN (2014). *Rhodotorula glutinis* as source of pigments and metabolites for food industry. Food Biosci.

[60] Botts MR, Giles SS, Gates MA, Kozel TR, Hull CM (2009). Isolation and characterization of *Cryptococcus neoformans* spores reveal a critical role for capsule biosynthesis genes in spore biogenesis. Eukaryot Cell.

[61] Maki LR, Galyan EL, Chang-Chien MM, Caldwell DR (1974). Ice nucleation induced by *Pseudomonas syringae*. Appl Microbiol.

[62] Morris CE, Sands DC, Vinatzer BA, Glaux C, Guilbaud C, Buffière A (2008). The life history of the plant pathogen *Pseudomonas syringae* is linked to the water cycle. ISME J.

[63] Hanlon R, Powers C, Failor K, Monteil CL, Vinatzer BA, Schmale DG (2017). Microbial ice nucleators scavenged from the atmosphere during simulated rain events. Atmos Environ.

[64] O’Sullivan D, Murray BJ, Ross JF, Webb ME (2016). The adsorption of fungal ice-nucleating proteins on mineral dusts: a terrestrial reservoir of atmospheric ice-nucleating particles. Atmos Chem Phys.

[65] O’Sullivan D, Murray BJ, Ross JF, Whale TF, Price HC, Atkinson JD (2015). The relevance of nanoscale biological fragments for ice nucleation in clouds. Sci Rep.

[66] Huffman JA, Prenni AJ, DeMott PJ, Pöhlker C, Mason RH, Robinson NH (2013). High concentrations of biological aerosol particles and ice nuclei during and after rain. Atmos Chem Phys.

[67] Dean R, Van Kan JAL, Pretorius ZA, Hammond-Kosack KE, Di Pietro A, Spanu PD (2012). The Top 10 fungal pathogens in molecular plant pathology. Mol Plant Pathol.

[68] Damm U, Fourie PH, Crous PW (2010). *Coniochaeta* (*Lecythophora*), *Collophora* gen. nov. and *Phaeomoniella* species associated with wood necroses of *Prunus* trees. Persoonia.

[69] Cech TL, Schwanda K, Klosterhuber M, Straßer L, Kirisits T (2016). Eutypella canker of maple: first report from Germany and situation in Austria. For Pathol.

[70] Pusz W, Król M, Zwijacz-Kozica T (2018). Airborne fungi as indicators of ecosystem disturbance: an example from selected Tatra Mountains caves (Poland). Aerobiologia.

