# Correction: Taxonomic diversity of fungi deposited from the atmosphere

**DOI:** 10.1038/s41396-019-0534-5

**Published:** 2019-10-21

**Authors:** Cheolwoon Woo, Choa An, Siyu Xu, Seung-Muk Yi, Naomichi Yamamoto

**Affiliations:** 1 Department of Environmental Health Sciences, Graduate School of Public Health, Seoul National University, Seoul, 08826 Republic of Korea; 2 Institute of Health and Environment, Seoul National University, Seoul, 08826 Republic of Korea

**Keywords:** Biogeochemistry, Ecosystem ecology, Climate-change impacts, Microbial ecology, Molecular ecology

**Correction to: The ISME Journal**

10.1038/s41396-018-0160-7

Since publication of our article, the authors have identified an error in Eq. (1). The correct equation is

1 $$V_d = \frac{{\mathop {\sum}\nolimits_{j = 1}^6 {F_j} }}{{\mathop {\sum}\nolimits_{j = 1}^6 {\mathop {\sum}\nolimits_{i = 1}^5 {N_{j,i}} } }}$$

All the reported results are calculated correctly by this equation. Therefore, there is no change in our reported values. The authors would like to apologize for any inconvenience caused by this error.

