# Supplementary material for: Taxonomic diversity of fungi deposited from the atmosphere
Source: ISME J. 2018 May 30;12(8):2051–60. doi: 10.1038/s41396-018-0160-7 (PMC6051994; doi:10.1038/s41396-018-0160-7)
Supplement: Supplementary file 1 — Supplementary Information [file 41396_2018_160_MOESM1_ESM.pdf]

Supplementary Information for:

Taxonomic diversity of fungi deposited from the atmosphere

Cheolwoon Woo [1†], Choa An [1†], Siyu Xu [1], Seung-Muk Yi [1,2], and Naomichi Yamamoto [1,2*]

[1] Department of Environmental Health Sciences, Graduate School of Public Health, Seoul National University, Seoul 08826, Republic of Korea

[2] Institute of Health and Environment, Seoul National University, Seoul 08826, Republic of Korea

[†] These authors contributed equally to this work

[*] Corresponding Author: Department of Environmental Health Sciences, Graduate School of Public Health, Seoul National University, Seoul 08826, Republic of Korea

Phone: +82-2-880-2837. E-mail: nyamamoto@snu.ac.kr

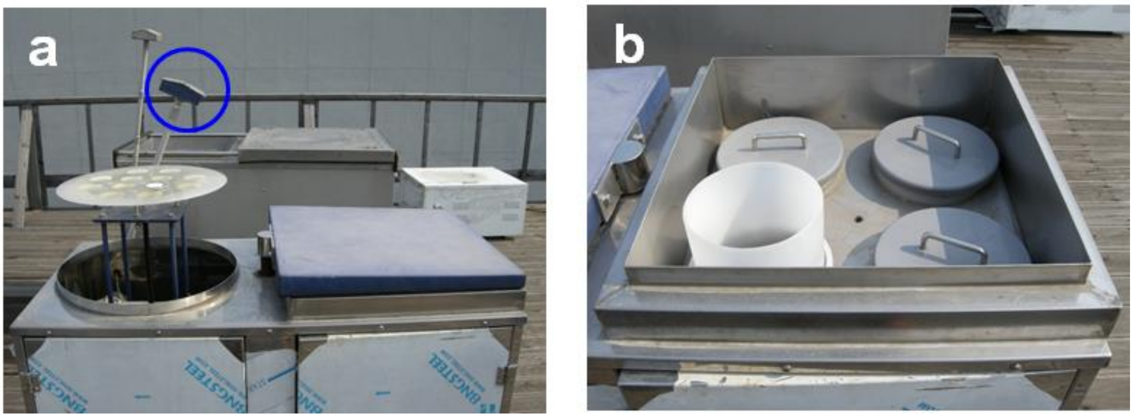

Supplementary Fig. S1 Automatic dry and wet deposition sampler. **a** Dry deposition samples are collected on 47-mm diameter substrates placed on the uplifted movable mount. **b** Wet deposition samples are collected in polypropylene bottles. The lid is automatically opened for wet deposition sampling and closed for dry deposition sampling when precipitation is detected by the precipitation sensor (circle). This means that dry deposition sampling is terminated during precipitation. Conversely, the lid is automatically opened for dry deposition sampling and closed for wet deposition sampling when precipitation is not sensed.

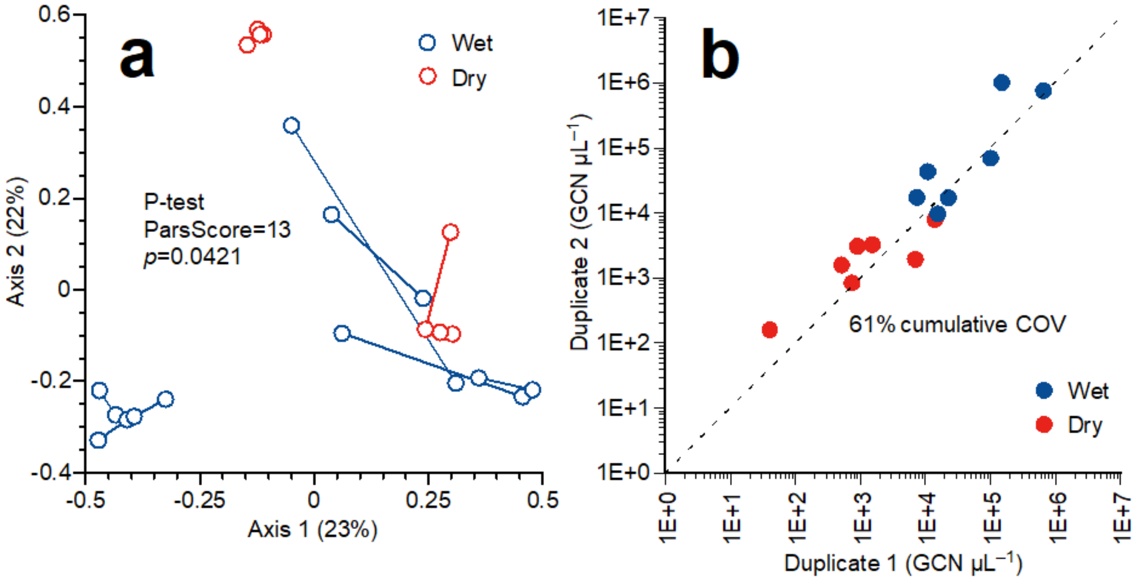

Supplementary Fig. S2 Reproducibility of biologically-duplicated fungal measurements. **a** Principal coordinate analysis plot for fungal assemblage structures in terms of Yue and Clayton theta similarity coefficients based on ITS1 OTUs at 97% sequence similarity. Each sample's duplicates are connected by a line. P-test shows the variability within a sample's duplicates is significantly smaller than the differences in fungal assemblage structures across the samples (ParScore=13, p=0.0421). **b** Fungal concentrations in DNA extracts quantitated by the universal fungal qPCR are shown, with 61% cumulative coefficient of variation (COV) (arithmetic scale) being confirmed for a series of the duplicated measurements. The dashed line represents 1:1.

Supplementary Table S1 Weather conditions during the sampling periods.

Month	Temperature (°C) [a]			Relative humidity (%) [a]			Wind velocity ($m\ s^{-1}$) [a]		Precipitation
	Highest [c]	Average	Lowest [c]	Highest [c]	Average	Lowest [c]	Highest [c]	Average	($mL\ cm^{-1}\ month^{-1}$) [b]
May 2015	32.2	18.9	8.3	90.4	52.8	27.1	6.4	3.0	3.7
June 2015	34.9	23.6	15.6	91.0	59.8	28.9	3.8	2.5	9.1
July 2015	36.0	25.8	18.2	96.9	71.2	49.8	5.2	2.8	18.6
August 2015	34.4	26.3	18.9	84.3	69.8	59.9	4.5	2.4	8.4
September 2015	31.0	22.4	14.2	79.8	56.2	36.6	3.7	2.3	2.6
October 2015	26.6	15.5	1.1	77.5	60.4	38.9	3.8	2.3	4.6
November 2015	21.0	8.9	–7.3	97.3	73.3	48.4	6	2.7	11.2

[a] Korea Meteorological Administration.

[b] This study.

[c] The values are based on 1-min monitoring interval.

Supplementary Table S2 Numbers and lengths of high-quality sequence reads by Illumina MiSeq.

Sampler	Start day (yymmdd)	End day (yymmdd)	Sample type	Duplicate	Sample ID	Number of sequences	Mean sequence length (bp)
Andersen	150501	150531	$d_a > 11$ μm	-	5A0	9689	282
			$d_a = 7–11$ μm	-	5A1	7428	279
			$d_a = 4.7–7$ μm	-	5A2	9449	284
			$d_a = 3.3–4.7$ μm	-	5A3	5464	291
			$d_a = 2.1–3.3$ μm	-	5A4	26055	309
	150601	150630	$d_a > 11$ μm	-	6A0	1196	314
			$d_a = 7–11$ μm	-	6A1	7927	281
			$d_a = 4.7–7$ μm	-	6A2	8425	296
			$d_a = 3.3–4.7$ μm	-	6A3	12626	299
			$d_a = 2.1–3.3$ μm	-	6A4	606	297
	150701	150731	$d_a > 11$ μm	-	7A0	12473	303
			$d_a = 7–11$ μm	-	7A1	10569	310
			$d_a = 4.7–7$ μm	-	7A2	10984	292
			$d_a = 3.3–4.7$ μm	-	7A3	1779	318
			$d_a = 2.1–3.3$ μm	-	7A4	32278	317
	150901	150930	$d_a > 11$ μm	-	9A0	5569	278
			$d_a = 7–11$ μm	-	9A1	15224	291
			$d_a = 4.7–7$ μm	-	9A2	15942	296
			$d_a = 3.3–4.7$ μm	-	9A3	12983	308
			$d_a = 2.1–3.3$ μm	-	9A4	27241	305
	151001	151031	$d_a > 11$ μm	-	10A0	7910	273
			$d_a = 7–11$ μm	-	10A1	9174	274
			$d_a = 4.7–7$ μm	-	10A2	4958	277
			$d_a = 3.3–4.7$ μm	-	10A3	9607	284
			$d_a = 2.1–3.3$ μm	-	10A4	13162	290
	151101	151130	$d_a > 11$ μm	-	11A0	8122	273
			$d_a = 7–11$ μm	-	11A1	10462	277
			$d_a = 4.7–7$ μm	-	11A2	3650	275
			$d_a = 3.3–4.7$ μm	-	11A3	7352	297
			$d_a = 2.1–3.3$ μm	-	11A4	22606	298
Deposition	150501	150531	dry	1	5D1	27834	283
			dry	2	5D2	20194	287
			wet	1	5W1	37973	290
			wet	2	5W2	31301	287
	150601	150630	dry	1	6D1	n.a.	n.a.
			dry	2	6D2	19968	294
			wet	1	6W1	23292	272
			wet	2	6W2	36893	276
	150701	150731	dry	1	7D1	18262	284
			dry	2	7D2	49419	281
			wet	1	7W1	7585	308
			wet	2	7W2	50889	307
	150801	150831	dry	1	8D1	23563	280
			dry	2	8D2	n.a.	n.a.
			wet	1	8W1	21278	280
			wet	2	8W2	35259	281
	150901	150930	dry	1	9D1	20477	276
			dry	2	9D2	9345	274
			wet	1	9W1	27623	287
			wet	2	9W2	8903	278
	151001	151031	dry	1	10D1	13535	273
			dry	2	10D2	4059	267

		wet	1	10W1	20946	282
		wet	2	10W2	32163	285
151101	151130	dry	1	11D1	21128	279
		dry	2	11D2	n.a.	n.a.
		wet	1	11W1	23400	303
		wet	2	11W2	43889	306

Symbol: -, duplicate not available.
Abbreviation: n.a., not PCR-amplifiable for sequencing.

Supplementary Table S3 Physical properties of selected fungal genera [a].

Phylum [b]	Class [b]	Genus [b]	Aerodynamic diameter (d_g, µm) [c]	Settling velocity (V_d, cm s^{-1})	Microscopy-based spore size in terms of diameter, length × width, or length × width × thickness [ref.]
Ascomycota	Dothideomycetes	*Alternaria*	10.0	0.63	18–83 × 7–18 µm for conidia of *Alternaria alternata* [1]
		Aureobasidium	9.14	13	17 µm for chlamydospores of *Aureobasidium pullulans* [2]
		Austroafricana	7.49	0.46	not found
		Cercospora	6.11	0.0063	not found
		Cladosporium	4.74	0.076	3–11 × 2–5 µm for conidia of *Cladosporium cladosporioides* [1]
		Curvularia	10.5	1.2	21–31 × 8.5–12 µm for conidia of *Curvularia lunata* [3]
		Didymella	6.49	1.1	12–16 × 4.5–6 µm for ascospores of *Didymella exigua* [4]
		Epicoccum	11.5	0.93	15–25 µm for conidia of *Epicoccum purpurascens* [1]
		Mycosphaerella	6.31	0.026	10–12 × 3–4 µm for ascospores of *Mycosphaerella alistairii* [5]
		Nothophoma	9.64	2.9	10–15 × 2.5–3 µm for conidia of *Nothophoma macrospora* [6]
		Paraconiothyrium	9.63	1.1	3.2–4.2 × 1.4–2 µm for conidia of *Paraconiothyrium estuarinum* [7]
		Periconia	9.72	0.46	7.5–9.5 µm diameter for globular conidia of *Periconia variicolor* [8]
		Selenophoma	10.1	64	17–22 × 2.7–3 µm for pycnospores of *Selenophoma linicola* [9]
		Stagonosporopsis	7.26	0.0011	3.5–5.5 × 1.5–3.5 µm for conidia of *Stagonosporopsis dennisii* [4]
		Stemphylium	11.1	0.46	22–26 × 14–16 µm for conidia of *Stemphylium beticola* [6]
		Trichomerium	9.82	0.77	20–22 × 5–7 µm for ascospores of *Trichomerium gloeosporum* [10]
	Eurotiomycetes	*Aspergillus*	5.13	0.063	2–3.5 µm for conidia of *Aspergillus fumigatus* [1]
		Penicillium	6.05	0.11	2.5–3 µm for conidia of *Penicillium citrinum* [1]
		Phaeococcomyces	12.0	0.18	3–5 × 2.5–5 µm for conidia of *Phaeococcomyces eucalypti* [11]
	Lecanoromycetes	*Ochrolechia*	11.5	0.38	47–55 × 19–31 µm for *Ochrolechia antillarum* [12]
	Leotiomycetes	*Botrytis*	7.17	0.018	7–11 × 5–6 µm for conidia of *Botrytis aclada* [1]
		Collophora	10.2	86	4.5–6.5 × 1–1.5 µm for conidia of *Collophora capensis* [13]
	Sordariomycetes	*Arthrinium*	7.02	0.027	7–9 µm for conidia of *Arthrinium sphaerospermum* [1]
		Chaetomium	4.82	0.30	7–8 × 5.5–6 × 4–5 µm for *Chaetomium afropilosum* [14]
		Gibberella	6.21	1.2	not found
		Nigrospora	9.93	0.67	12–16.5 × 9–15.5 µm for conidia of *Nigrospora aurantiaca* [15]
		Peroneutypa	6.07	0.0027	4.5–6.5 × 1–2 µm for ascospores of *Peroneutypa mackenziei* [16]
		Phomatospora	4.29	<0.001	14.5–17.8 × 5–6 µm for ascospores of *Phomatospora luteotingens* [17]
		Pseudovalsaria	5.71	0.014	11–14 × 4.5–5 µm for ascospores of *Pseudovalsaria foedans* [18]
		Trichoderma	7.39	0.23	2.8–4.3 × 2.1–2.4 µm for conidia of *Trichoderma hypoxylon* [19]
	Incertae sedis	*Alatosessilispora*	9.82	0.63	not found
		Knufia	9.03	2.0	9.5–16 × 8–14 µm for endoconidia of *Knufia tsunedae* [20]
Basidiomycota	Agaricomycetes	*Antrodia*	5.07	<0.001	5.9–8.2 × 2.4–3.2 µm for basidiospores of *Antrodia serialis* [21]
		Bjerkandera	3.45	0.010	3.0–5.0 × 1.2–2.2 µm for *Bjerkandera adusta* and 4.2–5 × 2.4–3.4 µm for *Bjerkandera fumosa* [22]

	Cabalodontia	3.74	<0.001	not found
	Coprinellus	4.78	0.0071	9.2–13.3 × 5.5–7 μm for basidiospores of *Coprinellus cinereopallidus* [23]
	Coprinopsis	5.33	0.012	11–16.5 × 6–8 μm for *Coprinopsis pseudomarcescibilis* [24]
	Coprinus	7.21	0.0072	13–20 × 8–12 μm for *Coprinus littoralis* [6]
	Daedaleopsis	4.99	0.035	not found
	Hyphodontia	3.70	0.019	5.8–6.8 × 1.4–1.8 μm for basidiospores of *Hyphodontia subalutacea* [25]
	Irpex	4.33	0.0022	5.5–6.5 × 3.5–4 μm for basidiospores of *Irpex hydnoides* [26]
	Junghuhnia	4.71	<0.001	2.1–2.6 × 1.5–2 μm for basidiospores of *Junghuhnia pseudominuta* and 2.7–3.0 × 1.9–2.1 μm for basidiospores of *Junghuhnia rhizomorpha* [27]
	Mycoacia	3.69	0.0052	not found
	Peniophora	5.12	0.034	6.5–8 × 3–3.5 μm for *Peniophora livida* [28]
	Peniophorella	4.77	<0.001	not found
	Perenniporia	4.70	0.015	4.6–5.8 × 3.1–3.8 μm for basidiospores of *Perenniporia narymica* [25]
	Phanerochaete	4.79	<0.001	4.7–5.3 × 2.5–3.1 μm for basidiospores of *Phanerochaete porostereoides* [29]
	Phellinus	3.76	<0.001	3.5 × 3 μm for basidiospores of *Phellinus lonicericola* [30]
	Phlebia	3.82	<0.001	3.5–4.4 × 2.1–2.8 μm for basidiospores of *Phlebia acanthocystis* [25]
	Phlebiopsis	4.35	0.0042	not found
	Psathyrella	4.57	0.040	7.1–8.2 × 3.2–4.1 μm for *Psathyrella koreana* [31]
	Schizophyllum	5.04	0.0018	not found
	Sistotrema	3.94	<0.001	5.7–6.5 × 3.3–4 μm for *Sistotrema epiphyllum* [32]
	Sistotremastrum	3.84	<0.001	5–6.5 × 2.5–3 μm for *Sistotremastrum guttuliferum* [33]
	Stereum	4.54	0.032	6–7 × 2.5 μm for *Stereum sanguinolentum* [34]
	Trametes	4.12	0.013	6.6–9.2 × 2.4–3 μm for *Trametes cystidiolophora* [35]
	Trechispora	3.91	0.052	not found
	Tyromyces	5.35	0.0013	3.7–5.0 × 2.1–3.0 μm for basidiospores of *Tyromyces kmetii* [36]
Dacrymycetes	*Cerinomyces*	5.25	0.011	7–12 × 3.5–5.5 μm for basidiospores of *Cerinomyces pallidus* [37]
Microbotryomycetes	*Rhodotorula* [d]	9.94	102	3–5 μm for cells of *Rhodotorula glutinis* [38]
Tremellomycetes	*Cryptococcus*	8.32	25	1–2 μm for spores and 5–10 μm for hydrated cells of *Cryptococcus neoformans* [39]
	Filobasidium	8.38	19	not found
	Hannaella	8.89	0.24	not found
Wallemiomycetes	*Wallemia*	4.98	0.23	2.5–3.5 μm for conidia of *Wallemia sebi* [1]

[a] The genera are selected if they were detected from all sampled months, each with more than 4 sequence reads from the samples of all particle size intervals combined.
[b] The classification is based on the Index Fungorum (http://www.indexfungorum.org/).
[c] Arithmetic means of d_g of all sampled months.
[d] *Rhodotorula* did not meet the abovementioned selection criteria, but included due to its importance of dry and wet deposition.

Supplementary Table S4 Taxonomic classification of the selected fungi reported by this study [a].

Phylum	Class	Order	Genus
Ascomycota	Dothideomycetes	Capnodiales	*Cladosporium*
			Arthrocatena
			Austroafricana
		Chaetothyriales	*Trichomerium*
		Dothideales	*Cylindroseptoria*
			Hormonema
			Aureobasidium
			Selenophoma
		Mycosphaerellales	*Cercospora*
			Mycosphaerella
		Pleosporales	*Didymella*
			Epicoccum
			Nothophoma
			Stagonosporopsis
			Periconia
			Paraconiothyrium
			Alternaria
			Curvularia
			Stemphylium
	Eurotiomycetes	Chaetothyriales	*Cyphellophora*
			Exophiala
			Phaeococcomyces
		Eurotiales	*Aspergillus*
			Penicillium
	Incertae sedis	*Incertae sedis*	*Alatosessilispora*
			Knufia
	Lecanoromycetes	Acarosporales	*Acarospora*
		Teloschistales	*Heterodermia*
		Pertusariales	*Ochrolechia*
	Leotiomycetes	Helotiales	*Botrytis*
		Phacidiales	*Collophora*
	Sordariomycetes	Hypocreales	*Trichoderma*
			Bisifusarium
			Fusarium
			Gibberella
		Incertae sedis	*Nigrospora*
		Phomatosporales	*Phomatospora*
		Coniochaetales	*Coniochaeta*
		Incertae sedis	*Arthrinium*
		Sordariales	*Chaetomium*
		Amphisphaeriales	*Pseudovalsaria*
		Xylariales	*Peroneutypa*
	Archaeorhizomycetes	Archaeorhizomycetales	*Archaeorhizomyces*
	Taphrinomycetes	Taphrinales	*Taphrina*

Basidiomycota	Agaricomycetes	Agaricales	*Coprinus*
			Coprinellus
			Coprinopsis
			Psathyrella
			Schizophyllum
		Cantharellales	*Sistotrema*
		Hymenochaetales	*Phellinus*
			Trichaptum
			Hyphodontia
		Incertae sedis	*Peniophorella*
		Polyporales	*Antrodia*
			Bjerkandera
			Cabalodontia
			Irpex
			Junghuhnia
			Mycoacia
			Phlebia
			Ceriporia
			Phanerochaete
			Phlebiopsis
			Porostereum
			Daedaleopsis
			Perenniporia
			Trametes
			Tyromyces
		Russulales	*Hericium*
			Peniophora
			Neoaleurodiscus
			Stereum
		Trechisporales	*Sistotremastrum*
			Trechispora
	Dacrymycetes	Dacrymycetales	*Cerinomyces*
	Tremellomycetes	Filobasidiales	*Filobasidium*
		Tremellales	*Hannaella*
			Cryptococcus
			Naganishia
			Papiliotrema
	Wallemiomycetes	Wallemiales	*Wallemia*
	Cystobasidiomycetes	Erythrobasidiales	*Erythrobasidium*
	Microbotryomycetes	Sporidiobolales	*Rhodotorula*
			Sporobolomyces

[a] The classification is based on the Index Fungorum (http://www.indexfungorum.org/).

Supplementary Table S5 Numbers of the sequences assigned to the top 50 most wanted fungi [40] [a].

Sample type	Sample ID	Number of sequences [b]				
		SH027064.07FU	SH493298.07FU	SH468151.07FU	SH455726.07FU	SH459716.07FU
Air	5A0	0	0	7	1	0
	5A1	0	0	6	1	0
	5A2	0	0	12	5	0
	5A3	0	0	0	2	6
	5A4	0	0	0	2	0
	6A0	0	0	0	0	0
	6A1	0	0	0	0	0
	6A2	0	0	2	1	0
	6A3	0	0	0	0	0
	6A4	0	0	0	0	0
	7A0	0	0	0	0	0
	7A1	0	0	0	0	0
	7A2	0	0	44	0	0
	7A3	0	0	0	0	0
	7A4	0	0	0	0	0
	9A0	0	0	4	0	0
	9A1	0	0	0	0	0
	9A2	0	0	0	0	0
	9A3	0	0	0	0	0
	9A4	0	0	0	2	0
	10A0	0	0	1	3	0
	10A1	0	0	6	0	0
	10A2	0	0	1	0	0
	10A3	1	0	3	6	0
	10A4	0	0	0	4	0
	11A0	0	0	0	0	0
	11A1	0	0	1	1	0
	11A2	0	0	0	0	0
	11A3	0	0	0	1	0
	11A4	0	0	0	8	0
Dry	5D1	0	1	0	0	0
	5D2	0	0	414	0	0
	6D1	n.a.	n.a.	n.a.	n.a.	n.a.
	6D2	0	1	0	0	0
	7D1	0	0	0	0	0
	7D2	0	0	503	0	0
	8D1	0	0	0	0	0
	8D2	n.a.	n.a.	n.a.	n.a.	n.a.
	9D1	0	0	22	0	0
	9D2	0	0	30	0	0
	10D1	0	0	36	12	0
	10D2	0	0	7	0	0
	11D1	0	0	0	0	0
	11D2	n.a.	n.a.	n.a.	n.a.	n.a.
Wet	5W1	0	0	17	0	0
	5W2	0	0	4	0	0
	6W1	0	0	7	0	0
	6W2	0	0	0	0	0
	7W1	0	0	0	0	0
	7W2	0	0	1	0	0
	8W1	0	0	1	0	0
	8W2	0	0	0	0	0
	9W1	0	0	5	0	0
	9W2	0	0	0	0	0
	10W1	0	0	44	0	0
	10W2	0	0	59	0	0
	11W1	0	0	2	0	0
	11W2	0	0	1	0	0

[a] The sequences were BLASTN-searched against the latest version of the fasta file containing the top 50 most wanted fungi, i.e., top50_release_01.12.2017.fasta.
[b] The numbers of the sequences aligned with the subject sequences with the aligned length longer than 250 bp and 100% identity are shown.
Abbreviation: n.a., The samples were not PCR-amplifiable for sequencing

Supplementary References

1. Cole GT, Samson RA (1984) The conidia. In: Al-Doory Y, Domson JF (ed). Mould allergy. Lea & Fibiger: Philadelphia, pp 66–104.
2. Wachowska U, Głowacka K, Mikołajczyk W, Kucharska K. Biofilm of *Aureobasidium pullulans* var. *pullulans* on winter wheat kernels and its effect on other microorganisms. Microbiology. 2016; 85:523–530.
3. Alex D, Li D, Calderone R, Peters SM. Identification of *Curvularia lunata* by polymerase chain reaction in a case of fungal endophthalmitis. Med Mycol Case Rep. 2013; 2:137–140.
4. Chen Q, Jiang JR, Zhang GZ, Cai L, Crous PW. Resolving the *Phoma enigma*. Stud Mycol. 2015; 82:137–217.
5. Crous P, Groenewald J. *Mycosphaerella alistairii*. Fungal Planet. 2006; 4.
6. Crous PW, Wingfield MJ, Richardson DM, Le Roux JJ, Strasberg D, Edwards J, et al. Fungal Planet description sheets: 400–468. Persoonia. 2016; 36:316–458.
7. Verkley G, Silva M, Wicklow D, Crous P. *Paraconiothyrium*, a new genus to accommodate the mycoparasite *Coniothyrium minitans*, anamorphs of *Paraphaeosphaeria*, and four new species. Stud Mycol. 2004; 4:323–335.
8. Cantrell SA, Hanlin RT, Emiliano A. *Periconia variicolor* sp. nov., a new species from Puerto Rico. Mycologia. 2007; 99:482–487.
9. Vanterpool TC. *Selenophoma linicola* sp. nov. on Flax in Saskatchewan. Mycologia. 1947; 39:341–348.
10. Hongsanan S, Tian Q, Hyde KD, Hu D-M. The asexual morph of *Trichomerium gloeosporum*. Mycosphere. 2016; 7:1473–1479.
11. Crous PW, Shivas RG, Wingfield MJ, Summerell BA, Rossman AY, Alves JL, et al. Fungal Planet description sheets: 128–153. Persoonia. 2012; 29:146–201.
12. Brodo IM. Studies in the lichen genus *Ochrolechia*. 2. *Corticolous* species of North America. Can J Bot. 1991; 69:733–772.
13. Damm U, Fourie PH, Crous PW. *Coniochaeta* (*Lecythophora*), *Collophora* gen. nov. and *Phaeomoniella* species associated with wood necroses of *Prunus* trees. Persoonia. 2010; 24:60–80.
14. Wang XW, Lombard L, Groenewald JZ, Li J, Videira SIR, Samson RA, et al. Phylogenetic reassessment of the *Chaetomium globosum* species complex. Persoonia. 2016; 36:83–133.
15. Wang M, Liu F, Crous PW, Cai L. Phylogenetic reassessment of *Nigrospora*: ubiquitous endophytes, plant and human pathogens. Persoonia. 2017; 39:118–142.
16. Shang Q-J, Hyde KD, Phookamsak R, Doilom M, Bhat DJ, Maharachchikumbura SSN, et al. *Diatrypella tectonae* and *Peroneutypa mackenziei* spp. nov. (Diatrypaceae) from northern Thailand. Mycol Prog. 2017; 16:463–476.
17. Fournier J, Lechat C. *Phomatospora luteotingens* sp. nov., a new aquatic species of *Phomatospora* from France and Spain. Mycosphere. 2010; 1:39–43.
18. Spooner BM. New or rare British microfungi from Esher Common, Surrey. Trans Br Mycol Soc. 1986; 86:401–408.
19. Sun J, Pei Y, Li E, Li W, Hyde KD, Yin W-B, et al. A new species of *Trichoderma hypoxylon* harbours abundant secondary metabolites. Sci Rep. 2016; 6:37369.

20. Crous PW, Wingfield MJ, Guarro J, Cheewangkoon R, van der Bank M, Swart WJ, et al. Fungal Planet description sheets: 154–213. Persoonia. 2013; 31:188–296.
21. Spirin V, Vlasák J, Miettinen O. Studies in the *Antrodia serialis* group (Polyporales, Basidiomycota). Mycologia. 2017; 109:217–230.
22. Jung PE, Fong JJ, Park MS, Oh S-Y, Kim C, Lim YW. Sequence validation for the identification of the white-rot fungi *Bjerkandera* in public sequence databases. J Microbiol Biotechnol. 2014; 24:1301–1307.
23. Nagy LG, Házi J, Vágvölgyi C, Papp T. Phylogeny and species delimitation in the genus *Coprinellus* with special emphasis on the haired species. Mycologia. 2012; 104:254–275.
24. Crous PW, Wingfield MJ, Burgess TI, Hardy G, Barber PA, Alvarado P, et al. Fungal Planet description sheets: 558–624. Persoonia. 2017; 38:240–384.
25. Jang Y, Jang S, Lee J, Lee H, Lim YW, Kim C, et al. Diversity of wood-inhabiting polyporoid and corticioid fungi in Odaesan National Park, Korea. Mycobiology. 2016; 44:217–236.
26. Lim YW, Jung HS. *Irpex hydnoides*, sp. nov. is new to science, based on morphological, cultural and molecular characters. Mycologia. 2003; 95:694–699.
27. Yuan HS, Dai YC. Two new species of *Junghuhnia* (Basidiomycota, Polyporales), and a key to the species of China. Nord J Bot. 2008; 26:96–100.
28. Whelden RM. A comparative study of basidia and cystidia in *Peniophora livida*. Am J Bot. 1936; 23:539–545.
29. Liu S, He S. *Phanerochaete porostereoides*, a new species in the core clade with brown generative hyphae from China. Mycosphere. 2016; 7:648–655.
30. Lee JS, Jung HS. Taxonomic study on Korean Aphyllophorales (5) - on some unrecorded genera and species. Mycobiology. 2006; 34:166–175.
31. Seok SJ, Kim YS, Kim WG, Kwon SW, Park IC. Notes on some new species of *Psathyrella*. Mycobiology. 2010; 38:323–327.
32. Crous PW, Wingfield MJ, Schumacher RK, Summerell BA, Giraldo A, Gené J, et al. Fungal Planet description sheets: 281–319. Persoonia. 2014; 33:212–289.
33. Telleria MT, Melo I, Dueñas M, Salcedo I, Beltrán-Tejera E, Rodríguez-Armas JL, et al. *Sistotremastrum guttuliferum*: a new species from the Macaronesian islands. Mycol Prog. 2013; 12:687–692.
34. Burt EA. The Thelephoraceae of North America. XII. *Stereum*. Ann Mo Bot Gard. 1920; 7:81–249.
35. Li H-J, Cui B-K. A new *Trametes* species from Southwest China. Mycotaxon. 2010; 113:263–267.
36. Park MS, Cho HJ, Kim NK, Park JY, Lee H, Park KH, et al. Ten new recorded species of macrofungi on Ulleung Island, Korea. Mycobiology. 2017; 45:286–296.
37. Shirouzu T, Hirose D, Tokumasu S. Taxonomic study of the Japanese *Dacrymycetes*. Persoonia. 2009; 23:16–34.
38. Hernández-Almanza A, Cesar Montanez J, Aguilar-González MA, Martínez-Ávila C, Rodríguez-Herrera R, Aguilar CN. *Rhodotorula glutinis* as source of pigments and metabolites for food industry. Food Biosci. 2014; 5:64–72.
39. Botts MR, Giles SS, Gates MA, Kozel TR, Hull CM. Isolation and characterization of *Cryptococcus neoformans* spores reveal a critical role for capsule biosynthesis genes in spore biogenesis. Eukaryot Cell. 2009; 8:595–605.

40. Nilsson RH, Wurzbacher C, Bahram M, R. M. Coimbra V, Larsson E, Tedersoo L, et al. Top 50 most wanted fungi. MycoKeys. 2016; 12:29–40.
